# Phytochemicals in MASLD: A Focused Review of Gut Microbiome‐Linked Mechanisms

**DOI:** 10.1002/ptr.70352

**Published:** 2026-04-21

**Authors:** Jeong In Seo, Su Min Kim, Hye Hyun Yoo

**Affiliations:** ^1^ Pharmacomicrobiomics Research Center and College of Pharmacy, Hanyang University Ansan Republic of Korea; ^2^ Skaggs School of Pharmacy and Pharmaceutical Sciences University of California San Diego La Jolla California USA

## Abstract

Metabolic dysfunction‐associated steatotic liver disease (MASLD) has emerged as a major global health burden, yet effective pharmacological options remain limited. Recent advances highlight the gut microbiome as a key modulator of liver metabolism, inflammation, and fibrosis, making it a promising therapeutic target. Among various non‐pharmacologic strategies, phytochemicals have drawn growing attention for their ability to influence the gut–liver axis through natural, multitarget mechanisms. This mini‐review summarizes preclinical and clinical evidence on phytochemicals that demonstrate metabolic benefits in MASLD, with a focus on their microbiome‐mediated effects. To this end, we classify these mechanistic pathways into three major continuums: restoration of gut microbial composition (causation), modulation of signaling mediators, i.e., gut microbial metabolites (mediation), and the resulting functional outcomes derived from these causal links (outcome). While early pre‐clinical data are encouraging, translation is challenged by issues such as mechanistic complexity, microbiome‐dependent heterogeneity, and regulatory ambiguity. Future studies incorporating multi‐omics analysis, mechanism‐linked trial designs, and stratified patient populations will be critical to advancing phytochemicals as safe, effective, and personalized interventions for MASLD.

**Abbreviations**
BAs: Bile acids
BSH: Bile salt hydrolases
CoA: Coenzyme A
DCA: deoxycholic acid
FGF15/19: fibroblast growth factor 15/19
FMT: Fecal microbiota transplantation
FXR: farnesoid X receptor
GCA: Glycocholic acid
LPS: Lipopolysaccharide
MASH: Metabolic dysfunction‐associated steatohepatitis
MASLD: Metabolic dysfunction‐associated steatotic liver disease
SCFAs: Short‐chain fatty acids
TCA: taurocholic acid
TGR5: Takeda G protein‐coupled receptor 5
TLR: Toll‐like receptor (TLR)
ZO‐1: zonula occludens‐1

## 1 Introduction

Metabolic dysfunction‐associated steatotic liver disease (MASLD) has emerged as the most prevalent chronic liver condition globally, affecting over 30% of the population and showing a rising trajectory in parallel with obesity and type 2 diabetes (Le et al. 2022). MASLD describes the presence of hepatic steatosis together with at least one metabolic risk factor and either no alcohol use or only minimal intake not expected to contribute to liver injury, whereas metabolic dysfunction‐associated steatohepatitis (MASH) reflects a more advanced condition defined by steatohepatitis in addition to at least one metabolic risk factor under the same alcohol‐use criteria (Cusi et al. 2025; Rinella et al. 2023). Although several agents have recently been approved for MASH, including resmetirom (Harrison et al. 2024), which has demonstrated only partial efficacy, and semaglutide (Boutari et al. 2026), no pharmacological therapy is currently approved for MASLD itself. Moreover, long‐term pharmacotherapy is often constrained by issues related to tolerability, cost, and accessibility. These limitations highlight the need for complementary or alternative strategies to support MASLD management.

Among such strategies, increasing attention has been directed toward dietary interventions and phytochemicals, which are naturally derived compounds found in plant‐based foods and traditional medicinal sources (Li and Weng 2017; Nwozo et al. 2023). These agents are of particular interest due to their low toxicity, diverse bioactivity, and potential for long‐term use (Li and Weng 2017). Several phytochemicals such as curcumin, resveratrol, and silymarin have been shown to alleviate MASLD by improving lipid metabolism, enhancing insulin sensitivity, and attenuating hepatic inflammation (Anushiravani et al. 2019; Chen et al. 2015; Cicero et al. 2020; Faghihzadeh et al. 2014; Kheong et al. 2017; Rahmani et al. 2016). However, their mechanisms of action are often multifaceted and not fully elucidated.

The pathophysiology of MASLD is closely linked to the gut–liver axis, in which the gut microbiota plays a central role (Anushiravani et al. 2019; Henao‐Mejia et al. 2012; Loomba et al. 2019). Disruptions in microbial composition (dysbiosis) can lead to increased intestinal permeability and translocation of endotoxins such as lipopolysaccharide (LPS), triggering hepatic inflammation via Toll‐like receptor (TLR) pathways (Kim et al. 2012; Thevaranjan et al. 2017; Xue et al. 2017). Altered microbial metabolism also affects key signaling molecules including short‐chain fatty acids (SCFAs) and bile acids (BAs), which in turn influence insulin sensitivity, lipid homeostasis, and fibrogenesis (Lau et al. 2025). A growing body of evidence suggests that phytochemicals may exert their hepatoprotective effects, at least in part, by modulating the gut microbiome and influencing these pathways. For example, changes in microbial diversity, SCFA production, BA profiles, or intestinal barrier function have been observed in animal models treated with various phytochemicals (Dong, Lou, Xu, and Wang 2025; Huang et al. 2023; Li et al. 2023; Liu, Cao, et al. 2021; Ma et al. 2024; Peng et al. 2025; Yan et al. 2024; Yu et al. 2024; Zhou et al. 2023). These microbiota‐mediated interactions represent a promising dimension of phytochemical action in the context of MASLD.

This mini‐review discusses phytochemicals that have been explored for their potential therapeutic relevance to MASLD and considers the possibility that interactions with the gut microbiome may contribute to their underlying mechanisms. We categorize their mechanisms into major microbial and metabolic pathways, highlight the translational potential, and discuss key challenges that must be addressed to integrate phytochemical strategies into MASLD therapy.

## 2 Mechanistic Pathways Linking Phytochemicals, the Microbiome, and Liver Metabolism

**FIGURE 1:**
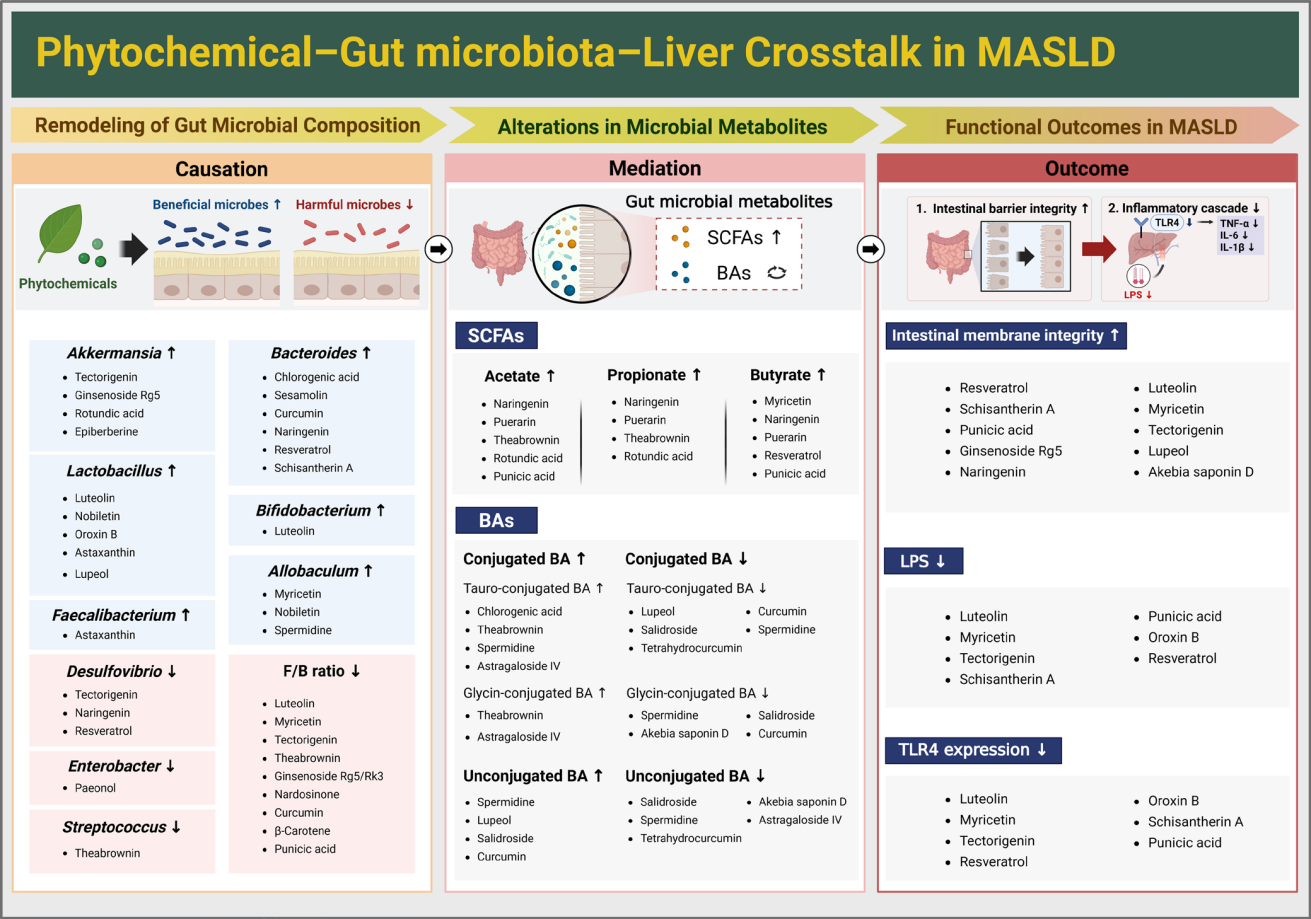
Schematic illustration of the phytochemical–gut microbiota–liver crosstalk in MASLD, outlining three major continuums (causation, mediation, and outcome). Causation: Phytochemicals can restore gut microbial dysbiosis associated with MASLD by rebalancing altered microbial profiles. Mediation: The reshaped gut microbiota modulates microbial metabolic pathways, resulting in altered metabolite profiles. These metabolites function as signaling molecules that mediate beneficial effects in MASLD. Outcome: Microbial metabolite–driven mechanisms contribute to MASLD improvement by (1) strengthening intestinal barrier integrity and (2) suppressing inflammatory cascades through reduced LPS translocation, thereby preventing TLR4 activation and subsequent proinflammatory cytokine release. Phytochemicals associated with each continuum are indicated within the respective boxes. Figure was created with BioRender.com.

**FIGURE 2:**
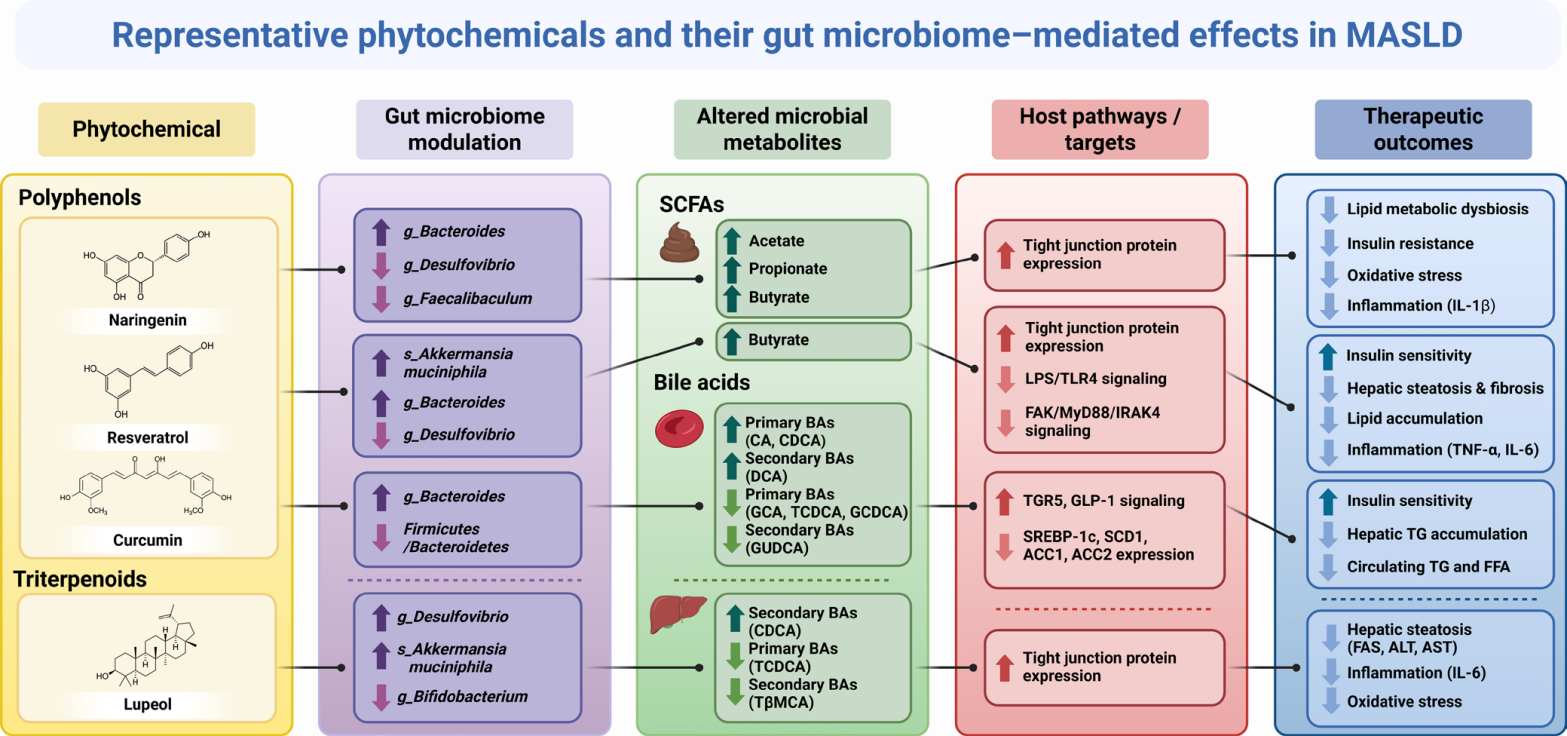
Representative phytochemicals and their gut microbiome mediated effects in MASLD. Four representative phytochemicals (resveratrol (Chen et al. 2020), naringenin (Dong, Lou, Xu, and Wang 2025), curcumin (He et al. 2024), and lupeol (Qin et al. 2024)) are highlighted to illustrate gut microbiome mediated therapeutic effects in MASLD across three conceptual continuums (causation, mediation, and outcome). Figure was created with BioRender.com.

Numerous phytochemicals have been shown to exert beneficial effects in MASLD preclinical models, and growing evidence suggests that their impact may be mediated through interactions with the gut microbiome. These interactions can be understood along a causal continuum comprising causation (restoration of gut microbial composition), which often leads to mediation (modulation of microbiota‐derived metabolites), and outcome (regulating MASLD‐relevant host functions, such as enhancing intestinal barrier integrity) (Figures 1 and 2). Furthermore, it is of particular interest that certain phytochemicals with low bioavailability, such as resveratrol (Wang et al. 2025) and myricetin (Park et al. 2016), which have limited intestinal absorption and/or extensive host metabolism (e.g., glucuronidation), may depend primarily on gut microbiota‐mediated mechanisms to exert therapeutic effects in MASLD. Hence, the low oral bioavailability traditionally regarded as a limitation in drug development due to insufficient systemic exposure (Choi et al. 2024; Kim et al. 2013; Seo et al. 2024) may warrant a different interpretation for phytochemicals whose therapeutic effects in MASLD are mediated through the gut microbiome. This suggests that limited absorption of such phytochemicals may not be inherently undesirable. Instead, prolonged luminal exposure, higher local concentrations in the gut, and reduced systemic adverse effects could represent a favorable pharmacokinetic profile rather than a liability.

In the following sections, we describe existing findings within each continuum and explain how each continuum can be causally linked to the next, thereby highlighting microbiome‐mediated mechanisms through which phytochemicals beneficially modulate MASLD pathophysiology.

### 2.1 Causation: Remodeling Gut Microbial Composition

Several microbial taxa are associated with MASLD severity. Especially, increased abundances of *Proteobacteria*, *Clostridium*, and *Streptococcus*, alongside reduced levels of beneficial bacteria such as *Eubacterium* and *Faecalibacterium*, have been reported (Lau et al. 2025). These dysbiotic changes contribute to disease progression by disrupting microbiome‐mediated mechanisms, whereas restoring the balance of these microbial populations may offer therapeutic benefits in MASLD.

Interestingly, several phytochemicals have been reported to exert therapeutic effects on MASLD by restoring gut dysbiosis, through increasing beneficial microbial taxa and/or reducing harmful ones in animal models (Table 1). These microbial shifts may underlie beneficial molecular mechanisms mediated by the gut microbiome, which are discussed in the following sections. For example, resveratrol treatment reversed HFD‐induced dysbiosis in mice, characterized by a decrease in the phylum *Firmicutes*, an increase in *Bacteroidetes*, a higher *Bacteroidetes*/*Firmicutes* ratio, and a reduction in the genus *Clostridium* (Pang et al. 2023). Also, epigallocatechin gallate treatment in a MASH mouse model (Ning et al. 2020) was strongly associated with changes in gut microbial composition, including an increase in *Alloprevotella* and *Bacteroides*, which showed a strong negative correlation with hepatic lipid accumulation, and a decrease in the pathogenic bacterium *Desulfovibrio* (Lin et al. 2022). Similarly, ginsenoside Rg5 and berberine alleviated MASLD by restoring gut dysbiosis (Shi et al. 2024; Shu et al. 2021). Notably, fecal microbiota transplantation (FMT) from ginsenoside Rg5‐treated mice with MASLD to MASLD recipient mice produced improvements comparable to those observed with direct ginsenoside Rg5 administration, suggesting that gut microbiota alterations may contribute to its beneficial actions (Shi et al. 2024).

**TABLE 1:** Key findings from preclinical and clinical studies on the gut microbiome–driven mechanisms underlying the therapeutic effects of phytochemicals in MASLD.

Study type	Class	Phytochemical	Changes in key microbes	Changes in gut microbial metabolites		Functional outcomes	Model type	References
				SCFA	BA			
Preclinical study	Polyphenols (Flavonoids)	Luteolin	↑ *p_Bacteroidetes*	N/A	N/A	↑ Tight junction protein expression	HFD Rats	(Liu, Cao, et al. 2021)
			↑ *g_Bifidobacterium*			↓ LPS levels		
			↑ *g_Desulfovibrio*			↓ TLR4 expression		
			↑ *g_Lactobacillus*					
			↓ *p_Firmicutes*					
			↓ *f_Desulfovibrionaceae*					
			↓ *Firmicutes/Bacteroidetes*					
		Myricetin	↑ *p_Firmicutes*	Feces	N/A	↑ Tight junction protein expression	HFD Rats	(Sun et al. 2021)
			↑ *g_Allobaculum*	↑ Butyrate		↓ LPS levels		
			↓ *Firmicutes/Bacteroidetes*	↓ Acetate		↓ TLR4 expression		
		Nobiletin	↑ *g_Allobaculum* (3)^a^	N/A	N/A	N/A	HFHS‐fed mice	(Li et al. 2023)
			↑ *g_Lactobacillus*					
			↓ *p_Firmicutes*					
			↓ *g_Allobaculum* (43)^a^					
		Tectorigenin	↑ *p_Bacteroidetes*	N/A	N/A	↑ Tight junction protein expression	HFD Rats	(Duan et al. 2022)
			↑ *f_Akkermansiaceae*			↓ LPS levels		
			↑ *g_Akkermansia*			↓ TLR4 expression		
			↓ *p_Firmicutes*					
			↓ *g_Desulfovibrio*					
			↓ *Firmicutes/Bacteroidetes*					
		Naringenin	↑ *p_Bacteroidetes*	Feces	N/A	↑ Tight junction protein expression	HFD mice	(Dong, Lou, and Wang 2025)
			↑ *f_Lactobacillaceae*	↑ Acetate				
			↑ *f_Ruminococcaceae*	↑ Propionate				
			↑ *g_Bacteroides*	↑ Butyrate				
			↓ *f_Desulfovibrionaceae*					
			↓ *g_Desulfovibrio*					
			↓ *g_Faecalibaculum*					
		Oroxin B	↑ *g_Lactobacillus*	N/A	N/A	↓ LPS levels	HFD Rats	(Huang et al. 2023)
						↓ TLR4 expression		
		Puerarin	↑ *p_Bacteroidetes*	Intestine	N/A	N/A	HFD mice	(Sun et al. 2025)
			↓ *p_Firmicutes*	↑ Acetate				
				↑ Propionate				
				↑ Butyrate				
	Polyphenols (Stilbenes)	Resveratrol	↑ *f_Ruminococcaceae*	Feces	N/A	↑ Tight junction protein expression	HFD Rats	(Chen et al. 2020)
			↑ *g_Bacteroides*	↑ Butyrate		↓ LPS levels		
			↑ *s_Akkermansia muciniphila*			↓ LPS/TLR4 signaling pathways		
			↓ *g_Desulfovibrio*					
	Polyphenols (Phenolic acids)	Chlorogenic acid	↑ *g_Bacteroides*	N/A	Cecum	N/A	HFD mice	(Yu et al. 2024)
					↑ Primary BAs (THCA, TCA)			
	Polyphenols (Diarylheptanoid)	Tetrahydrocurcumin	↑ *p_Bacteroidetes*	N/A	Serum	N/A	MCD diet‐induced MASH mice	(Peng et al. 2025)
			↑ *p_Desulfobacterota*		↓ Primary BAs (β‐MCA, CA)			
			↑ *f_Akkermansiaceae*		↓ Secondary BAs (23‐DCA, UCA, 7‐KDCA, HDCA, TDCA)			
			↑ *f_Desulfovibrionaceae*					
			↑ *Firmicutes/Bacteroidetes*					
			↓ *p_Firmicutes*					
	Polyphenols (Lignans)	Sesamolin	↑ *g_Bacteroides*	N/A	N/A	N/A	HF–HF diet fed mice	(Yu, Sun, et al. 2022)
			↓ *f_unclassified_Desulfovibrionaceae*					
			↓ *g_Allobaculum*					
			↓ *g_Faecalibaculum*					
		Schisantherin A	↑ *p_Bacteroidetes*	N/A	N/A	↑ Tight junction protein expression	HFD mice	(Yu, Sun, et al. 2022)
			↑ *c_Bacteroidia*			↓ LPS levels		
			↑ *g_Bacteroides*			↓ TLR4 expression		
			↓ *p_Firmicutes*					
	Polyphenols (Tea polyphenol)	Theabrownin	↓ *p_Firmicutes*	Intestine	Liver and ileum	N/A	HFD mice	(Chen, Lei, et al. 2024)
			↓ *g_Enterococcus*	↑ Acetate	↑ Primary BA (TCDCA)			
			↓ *g_Faecalibaculum*	↑ Propionate	↑ Secondary BA (TUDCA)			
			↓ *g_Lactobacillus*					
			↓ *g_Streptococcus*					
			↓ *Firmicutes/Bacteroidetes*					
	Monophenols	Paeonol	↑ *p_Bacteroidetes*	N/A	N/A	N/A	MCD high‐fat diet mice	(Yan et al. 2024)
			↓ *g_Enterobacter*					
	Polyamines (Aliphatic polyamines)	Spermidine	↑ *p_Bacteroidetes*	N/A	Liver	N/A	WD mice	(Ni et al. 2022)
			↑ *g_Allobaculum*		↑ Primary BAs (CA, α‐MCA, β‐MCA, TαMCA, TβMCA, CA/CDCA)			
			↓ *p_Firmicutes*		↓ Secondary BAs (DCA, GDCA, TDCA, TLCA, GDCA/CA, TDCA/CA)			
	Terpenoids (Carotenoids)	β‐Carotene	↓ *Firmicutes/Bacteroidetes*	N/A	N/A	N/A	HFD mice	(Dong, Lou, and Wang 2025)
		Astaxanthin	↑ g_*Faecalibacterium*	N/A	N/A	N/A	HFD mice	(Li et al. 2022)
			↑ *g_Lactobacillus*					
			↑ *g_Lactococcus*					
			↓ *f_Desulfovibrionaceae*					
	Triterpenoids (Lupanes)	Lupeol	↑ *p_Bacteroidetes*	N/A	Liver	↑ Tight junction protein expression	HFD mice	(Qin et al. 2024)
					↑ Secondary BA (CDCA)			
			↑ *g_Lactobacillus*		↓ Bound/unbound BA			
					↓ Primary BA (TDCA)			
			↑ *s_Akkermansia muciniphila*		↓ Secondary BA (TβMCA)			
			↓ *p_Firmicutes*		Feces			
			↓ *g_Bifidobacterium*		↑ Primary BA (MCA)			
	Triterpenoids (Saponins)	Ginsenoside Rg5	↑ *g_Akkermansia*	N/A	N/A	↑ Tight junction protein expression	HFD mice	(Shi et al. 2024)
			↓ *p_Firmicutes*					
			↓ *g_Allobaculum*					
			↓ *g_Bacteroides*					
			↓ *Firmicutes/Bacteroidetes*					
		Ginsenoside Rk3	↑ *f_Akkermansiaceae*	Feces	N/A	N/A	HFHC diet mice	(Guo et al. 2023)
			↓ *p_Firmicutes*	↓ Acetate				
			↓ *g_Bacteroides*	↓ Propionate				
			↓ *g_Faecalibaculum*	↓ Isobutyrate				
			↓ *Firmicutes/Bacteroidetes*					
		Astragaloside IV	↓ *g_Allobaculum*	N/A	Ileum	N/A	HFD mice	(Zhai et al. 2022)
			↓ *g_Bacteroides*		↑ Primary BAs (GCDCA, TβMCA)			
			↓ *g_Enterococcus*		↑ Secondary BAs (TUDCA, TDCA)			
			↓ *g_Lactobacillus*		↓ Unconjugated/conjugated BA			
			↓ *g_Lactococcus*		↓ Primary BAs (CDCA, CA, ωMCA)			
					↓ Secondary BA(DCA)			
		Akebia saponin D	↑ *f_Bifidobacteriaceae*	N/A	Feces	↑ Tight junction protein expression	HFD mice	(Yang et al. 2021)
			↑ *f_Ruminococcaceae*		↓ Primary BAs (CA, GCDCA, HDCA)			
	Triterpenoids (Pentacyclic triterpenoids)	Rotundic acid	↑ *g_Akkermansia*	Feces	N/A	N/A	HFD mice	(Li et al. 2021)
			↑ *g_Desulfovibrio*	↑ Acetate				
				↑ Propionate				
	Terpenes (Sesquiterpenes)	Nardosinone	↑ *g_Ruminococcaceae*_UCG‐014	N/A	N/A	N/A	HFD mice	(Ma et al. 2024)
			↑ *s_Enterococcus_casseliflavus*					
			↓ *g_Enterococcus*					
			↓ *g_Lactobacillus*					
			↓ *s_Enterococccus_faecalis*					
			↓ *Firmicutes/Bacteroidetes*					
	Alkaloids (Isoquinoline alkaloids)	Epiberberine	↑ *g_Akkermansia*	N/A	N/A	N/A	MCD mice	(Zhou et al. 2023)
	Fatty Acids (Polyunsaturated fatty acid)	Punicic acid	↑ *p_Bacteroidota*	Feces	N/A	↑ Tight junction protein expression	HFHF diet mice	(Chen, Ho, et al. 2024)
			↓ *p_Desulfobacterota*	↑ Acetate		↓ LPS levels		
			↓ *p_Firmicutes*	↑ Butyrate		↓ TLR4 expression		
			↓ *Firmicutes/Bacteroidetes*					
	Phenylethanoids (Phenylpropanoid glycosides)	Salidroside	↑ *g_Ruminoclostridium*	N/A	Colon	N/A	HFD mice	(Li et al. 2020)
			↓ *g_Lactobacillus*		↑ Secondary BAs (βCDCA, isoLCA, 12‐ketoLCA)			
					↓ Primary BAs (GCA, TCA, TCDCA, TβMCA, TαMCA)			
					↓ Secondary BAs (DCA, GDCA)			
	Amino acids (Branched‐chain amino acids)	Valine	↑ *s_Bacteroides cellulosilyticus*	N/A	N/A	N/A	HFD mice	(Felicianna et al. 2024)
	Polysaccharide‐based compound	Selenium polysaccharides	↑ *g_Akkermansia*	N/A	Feces	↑ Tight junction protein expression	HFD mice	(Luo et al. 2024)
			↑ *g_Bifidobacterium*		↓ Primary BAs (GCA, TβMCA)	↓ LPS levels		
			↓ *g_Desulfovibrio*		↓ Secondary BA (TDCA)			
			↓ *g_Roseburia*					
Clinical study	Polyphenols (Tea polyphenol)	Theabrownin	↓ *p_Firmicutes*	N/A	Serum	↓ BSH activity	Healthy male	(F. Huang et al. 2019)
			↓ *g_Faecalibaculum*		↑ Primary BA(GCDCA)			
			↓ *g_Lactobacillus*		↑ Secondary BA(GUDCA)			
			↓ *g_Streptococcus*					
			↓ *Firmicutes/Bacteroidetes*					
	Polyphenols (Diarylheptanoid)	Curcumin	↑ *g_Bacteroides*	N/A	Serum	↑ TGR5	MASLD patients	(He et al. 2024)
			↓ *p_Firmicutes*		↑ Primary BAs (CA, CDCA)	↑ GLP‐1		
			↓ *Firmicutes/Bacteroidetes*		↑ Secondary BA (DCA)			
					↑ Secondary/primary BAs			
					↓ Primary BAs (GCA, TCDCA, GCDCA)			
					↓ Secondary BA (GUDCA)			
					↓ Conjugated/unconjugated BAs			

Abbreviations: 12‐ketoLCA, 12‐ketolithocholic acid; 23‐DCA, nor‐deoxycholic acid; 7‐KDCA, 7‐ketodeoxycholic acid; BA, bile acid; BSH, bile salt hydrolase; CA, cholic acid; CDCA, chenodeoxycholic acid; DCA, deoxycholic acid; GCA, glycocholic acid; GCDCA, glycochenodeoxycholic acid; GUDCA, glycoursodeoxycholic acid; HDCA, hyodeoxycholic acid; HFD, high‐fat diet; HFHC, high‐fat‐high‐cholesterol; HF–HF, high fat and high fructose; HFHS, high‐fat/high‐sucrose; IAA, indole‐3‐acetic acid; isoLCA, isolithocholic acid; LPS, lipopolysaccharide; MCA, muricholic acid; MCD, methionine‐choline‐deficiency; N/A, Not applicable; SCFA, short‐chain fatty acid; TCDCA, taurochenodeoxycholic acid; TDCA, taurodeoxycholic acid; THCA, taurohyocholic acid; TLCA, taurolithocholic acid; TLR 4, toll‐like receptor 4; TUDCA, tauroursodeoxycholic acid; TαMCA, tauro α‐muricholic acid; TβMCA, tauro β‐muricholic acid; UCA, ursodeoxycholic acid; WD, western diet; α‐MCA, α‐muricholic acid; βCDCA, 3β‐chenodeoxycholic acid; β‐MCA, β‐muricholic acid; ωMCA, ω‐muricholic acid.

^a^ The number of altered genera is shown in parentheses.

Furthermore, the impact of phytochemicals on gut microbial composition may be, at least in part, compound‐specific as shown in animal models. For example, chlorogenic acid has been reported to enrich *Akkermansia muciniphila* , a microbe often linked to host‐protective effects such as enhanced gut barrier function (Cani et al. 2022), partly through the suppression of pathogenic genera such as *Desulfovibrio* and *Alistipes* (Everard et al. 2013; Gao et al. 2024; Wang et al. 2022). In contrast, a study showed that resveratrol was associated with a reduction in *Akkermansia* levels, while still conferring therapeutic benefits (Sung et al. 2017). These divergent microbial responses underscore the potential utility of phytochemical combinations to broaden the scope of microbial modulation. Indeed, co‐administration of resveratrol and quercetin has demonstrated synergistic effects in ameliorating high‐fat diet (HFD)‐induced obesity through restoration of gut microbial balance in a preclinical model (Zhao et al. 2017).

### 2.2 Mediation: Altering Microbial Metabolites to Modulate the Gut–Liver Axis

Microbial metabolites are closely linked to the pathophysiology of MASLD, functioning as key signaling molecules (Lau et al. 2025). Among these metabolites, SCFAs and BAs are the most extensively studied as mediators in ameliorating MASLD (Schnabl et al. 2025), and both have been shown to be modulated by phytochemical interventions (Chen et al. 2020; Dong, Lou, and Wang 2025; He et al. 2024; Huang et al. 2019; Ni et al. 2022; Qin et al. 2024; Sun et al. 2025; Zhai et al. 2022). The following sections describe the causative links by which phytochemical‐induced changes in gut microbial composition lead to the modulation of these molecular mediators.

#### 2.2.1 Enhancement of Short‐Chain Fatty Acids (SCFAs)

Alterations in gut microbial taxa following phytochemical intervention can modulate the production of gut microbial metabolites that mediate therapeutic effects via the gut–liver axis. Notably, MASLD patients often show reduced levels of SCFAs, particularly acetate and butyrate, due to a decreased abundance of SCFA‐producing bacteria like *Faecalibacterium* and *Roseburia* (C. Yang et al. 2024), with several phytochemicals reported to modulate SCFA levels in preclinical models (Chen, Ho, et al. 2024; Sun et al. 2021). Given their established roles such as maintaining intestinal barrier integrity and stimulating GLP‐1 secretion through SCFA–GPR41/GPR43 signaling, diminished SCFA levels are thought to contribute to the pathophysiology of MASLD (Greiner and Backhed 2016; Martin‐Gallausiaux et al. 2021).

Indeed, various phytochemicals have been shown to restore the reduced levels of SCFAs in MASLD (Table 1). Resveratrol, a natural polyphenol that belongs to the stilbene class, increased the levels of butyrate when it is fed at high dose (100 mg/kg) to HFD‐fed rats with the increase of butyrate producing bacteria (e.g., *Ruminococcacaea* and *Lachnospiraceae*) (Chen et al. 2020). Similarly, several flavonoids increased SCFAs in MASLD animal models. For example, myricetin treatment in rats with MASLD significantly increased fecal butyrate levels, along with an increase in butyrate‐producing bacteria and expression of butyrate synthesis‐related genes (Sun et al. 2021). Interestingly, acetate levels were reduced, even compared to MASLD rats without myricetin treatment, likely because butyrate can be synthesized from acetate and the responsible enzyme (butyryl coenzyme A (CoA):acetate‐CoA transferase (Trachsel et al. 2016)) was also upregulated. Another flavonoid, puerarin, showed distinct effects on SCFA metabolism in MASLD mice, increasing acetate and propionate levels without altering butyrate, along with shifts in gut microbial composition (Sun et al. 2025). These varying results highlight the complex regulatory mechanisms by which individual phytochemicals influence SCFA metabolism. Accordingly, they underscore the potential for personalized phytochemical interventions tailored to specific microbial and metabolic profiles in MASLD.

#### 2.2.2 Regulation of Bile Acid Metabolism

Distinct gut microbiome profiles associated with MASLD severity influence BA metabolism through microbial enzymes such as bile salt hydrolases (BSH), which contribute to the conversion of primary BAs into secondary BAs, thereby altering the overall BA pool composition (Smirnova et al. 2022). Notably, a cohort study demonstrated that individuals with MASLD show increased circulating levels of specific BAs, including primary BAs such as glycocholic acid (GCA) and taurocholic acid (TCA), as well as the secondary BA, deoxycholic acid (DCA) (Rivera‐Andrade et al. 2022). These alterations in BA composition may contribute to MASLD pathophysiology via modulating the gut‐liver axis, as BAs act as signaling molecules that interact with host receptors, most notably farnesoid X receptor (FXR) and Takeda G protein‐coupled receptor 5 (TGR5), and trigger signaling cascades such as the FXR–fibroblast growth factor 15/19 (FGF15/19) axis (Gadaleta and Moschetta 2019) to modulate glucose and lipid metabolism, inflammation, and intestinal barrier integrity (Chiang and Ferrell 2020; Song et al. 2025).

These beneficial downstream effects have been demonstrated with several phytochemicals in MASLD (Table 1). Notably, theabrownin, a principal bioactive component of Pu‐erh tea, a widely consumed Chinese tea, was shown to suppress BSH‐producing bacterial genera, including *Lactobacillus*, *Bacillus*, *Streptococcus*, *Lactococcus*, and *Enterococcus* (Huang et al. 2019). This suppression led to a reduction in BSH activity and a subsequent increase in conjugated BAs (e.g., tauro‐chenodeoxycholic acid and tauro‐ursodeoxycholic acid, as well as total BA levels in the ileum of HFD‐fed mouse). The resulting modulation of gut microbiota–encoded BA metabolism attenuated intestinal FXR–FGF15 signaling, which in turn promoted hepatic BA synthesis, including activation of the alternative pathway, through the upregulation of enzymes such as CYP27A1 and CYP7B1. Ultimately, this cascade contributed to the activation of hepatic lipolysis. Similarly, astragaloside IV, a triterpenoid saponin, has been shown to reduce BSH activity by suppressing BSH‐producing microbial populations, thereby lowering the ratio of unconjugated to conjugated BAs in HFD‐fed mouse (Zhai et al. 2022). This shift in BA metabolism notably increases intestinal levels of tauro‐β‐muricholic acid, an antagonist of intestinal FXR (Sayin et al. 2013). The resulting inhibition of intestinal FXR leads to enhanced GLP‐1 secretion and reduced FGF15 expression, contributing to the amelioration of hepatic steatosis. Interestingly, in contrast to theabrownin, which notably promotes alternative BA metabolism in the liver by modulating the gut microbiome and its BA metabolic mechanisms, another phytochemical, spermidine, a naturally occurring polyamine, appears to act through a distinct mechanism (Ni et al. 2022). In obese mice, spermidine treatment ameliorated MASH by significantly altering hepatic BA profiles, notably decreasing DCA and increasing the cholic acid/chenodeoxycholic acid ratio, suggestive of a more pronounced activation of classical BA synthesis (Ni et al. 2022). Notably, this shift occurred despite the suppression of BSH‐producing bacteria such as *Blautia* (Z. Song et al. 2019) highlighting mechanistic divergence among phytochemicals in modulating the gut–BA axis to alleviate MASLD.

### 2.3 Outcome: Potential Effects on Restoring Host Barrier and Immune Homeostasis

Phytochemical‐induced modulation of gut microbial composition alters microbial metabolite profiles, leading to functional effects that have been shown to be associated with MASLD improvement in various preclinical models. In particular, phytochemicals may counteract the inflammatory cascade driven by increased intestinal permeability and the subsequent influx of harmful microbial products into the liver (Miele et al. 2009), as discussed in the following section.

#### 2.3.1 Improvement of Gut Barrier Integrity and Endotoxemia

Gut dysbiosis compromises intestinal barrier integrity, resulting in increased permeability that facilitates the translocation of harmful microbial products into the portal circulation (Albillos et al. 2020). This hallmark feature of MASLD may contribute to endotoxemia, a condition characterized by the presence of LPS in the bloodstream (Belancic 2020). Notably, enhanced expression of tight junction proteins such as occludin, claudin‐1, and zonula occludens‐1 (ZO‐1) has been associated with improved intestinal barrier integrity (Gonzalez‐Mariscal et al. 2003). Accumulating preclinical evidence further suggests that phytochemical‐induced modulation of gut microbial composition and microbial metabolite profiles has been observed together with enhanced tight junction protein expression, and these changes may collectively influence host barrier function through microbiome‐mediated mechanisms.

To date, several studies have clearly demonstrated this functional outcome in conjunction with phytochemical‐induced alterations in gut microbial composition and metabolites (Table 1). For example, myricetin supplementation in HFD‐fed rats restored gut microbial composition and increased butyrate levels, leading to the upregulation of the tight junction protein ZO‐1 in the colon, whose expression is typically downregulated in this disease model (Sun et al. 2021). This enhancement of intestinal barrier integrity through gut microbiota modulation has also been demonstrated by many other phytochemicals, including luteolin (Liu, Cao, et al. 2021) and schisantherin A (Yu, Jiang, et al. 2022), and represents a key mechanism by which they exert therapeutic effects against MASLD. By improving intestinal barrier integrity, which may help limit systemic LPS influx, these phytochemicals indirectly modulate hepatic inflammatory responses, as further detailed in the following section.

#### 2.3.2 Suppression of Inflammatory Pathways

The leakage of microbial products, a downstream effect of impaired barrier integrity, leads to systemic endotoxemia and fuels chronic low‐grade inflammation associated with MASLD (An et al. 2022; Belancic 2020). Mechanistically, LPS activates hepatic Kupffer cells via the Toll‐like receptor 4 (TLR4) signaling pathway, inducing the production of pro‐inflammatory cytokines such as TNF‐α, IL‐6, and IL‐1β, which could contribute to the progression of MASLD (Koyama and Brenner 2017). Accordingly, preclinical studies suggest that attenuation of this microbiome‐associated inflammatory pathway may represent a potential avenue for limiting disease progression in MASLD.

Previous studies have demonstrated that phytochemicals such as luteolin (Liu, Sun, et al. 2021), myricetin (Sun et al. 2021), tectorigenin (Duan et al. 2022), schisantherin A (Yu, Sun, et al. 2022), and puerarin (Sun et al. 2025) enhance intestinal barrier integrity, an effect triggered by gut microbiome modulation (Table 1). This microbiota‐driven improvement in barrier function was associated with reduced LPS levels, which in turn contributed to the downregulation of TLR‐4 expression, suppression of NF‐κB activation, and decreased production of pro‐inflammatory cytokines, including IL‐1β, IL‐6, and TNF‐α (Lu et al. 2008). Ultimately, the microbiome‐driven effects of phytochemicals alleviate MASLD by modulating key indicators, such as liver injury markers, lipid profiles, hepatic steatosis, and insulin resistance, highlighting the significance of microbiome involvement in their therapeutic action.

## 3 Clinical Evidence of Microbiome‐Linked Effects of Phytochemicals

At this point, evidence supporting the phytochemical–gut microbiome–MASLD axis in humans remains limited, making it difficult to directly translate outcomes from preclinical models to clinical settings due to interspecies differences in microbiome composition (Chung et al. 2012). Nonetheless, the substantial functional redundancy between animal models and humans, for example, the mouse gut microbiome shares approximately 95.2% functional similarity with that of humans (Xiao et al. 2015), suggests that key mechanistic insights may still be translatable.

While limited in number, several studies have nonetheless demonstrated the beneficial effects of phytochemicals on MASLD via modulation of the gut microbiota (Table 1). In line with preclinical findings (Feng et al. 2019), the involvement of gut microbiota was also evident in clinical settings. A 24‐week intervention with curcumin (500 mg/day) in MASLD patients (*n* = 40) resulted in a significant reduction in hepatic fat content, accompanied by modulation of gut microbiota composition, characterized by a decrease in *Proteobacteria*, an increased *Firmicutes*‐to‐*Bacteroidetes* ratio, and enrichment of *Bacteroides*, as well as regulation of microbiota‐derived BA profiles, including elevated levels of DCA in serum (He et al. 2024). Similarly, silymarin, a widely used milk thistle–derived phytochemical with hepatoprotective effects (Abenavoli et al. 2018), has been investigated in MASLD, particularly in relation to gut microbiota involvement. In a 24‐week intervention involving 41 MASLD patients, silymarin treatment resulted in a significant reduction in liver stiffness, along with increased gut microbial diversity and compositional shifts (Jin et al. 2024). Notably, enrichment of *Oscillospiraceae*, a SCFA–producing bacterial family negatively associated with various disease states (Chen, Xie, et al. 2024), was observed. Despite its low bioavailability (Loguercio and Festi 2011), the observed effects of silymarin suggest that modulation of the gut microbiome may be a key mechanism through which it exerts its therapeutic benefits (Loguercio and Festi 2011). Taken together, the current findings and their derived implications underscore the need for future human studies to better elucidate the role of the gut microbiome in mediating the MASLD‐ameliorating potential of diverse phytochemicals.

## 4 Future Perspectives: Unlocking the Potential of Microbiome‐Targeting Phytochemicals

### 4.1 Unique Opportunities of Phytochemicals Targeting the Gut–Liver Axis

**FIGURE 3:**
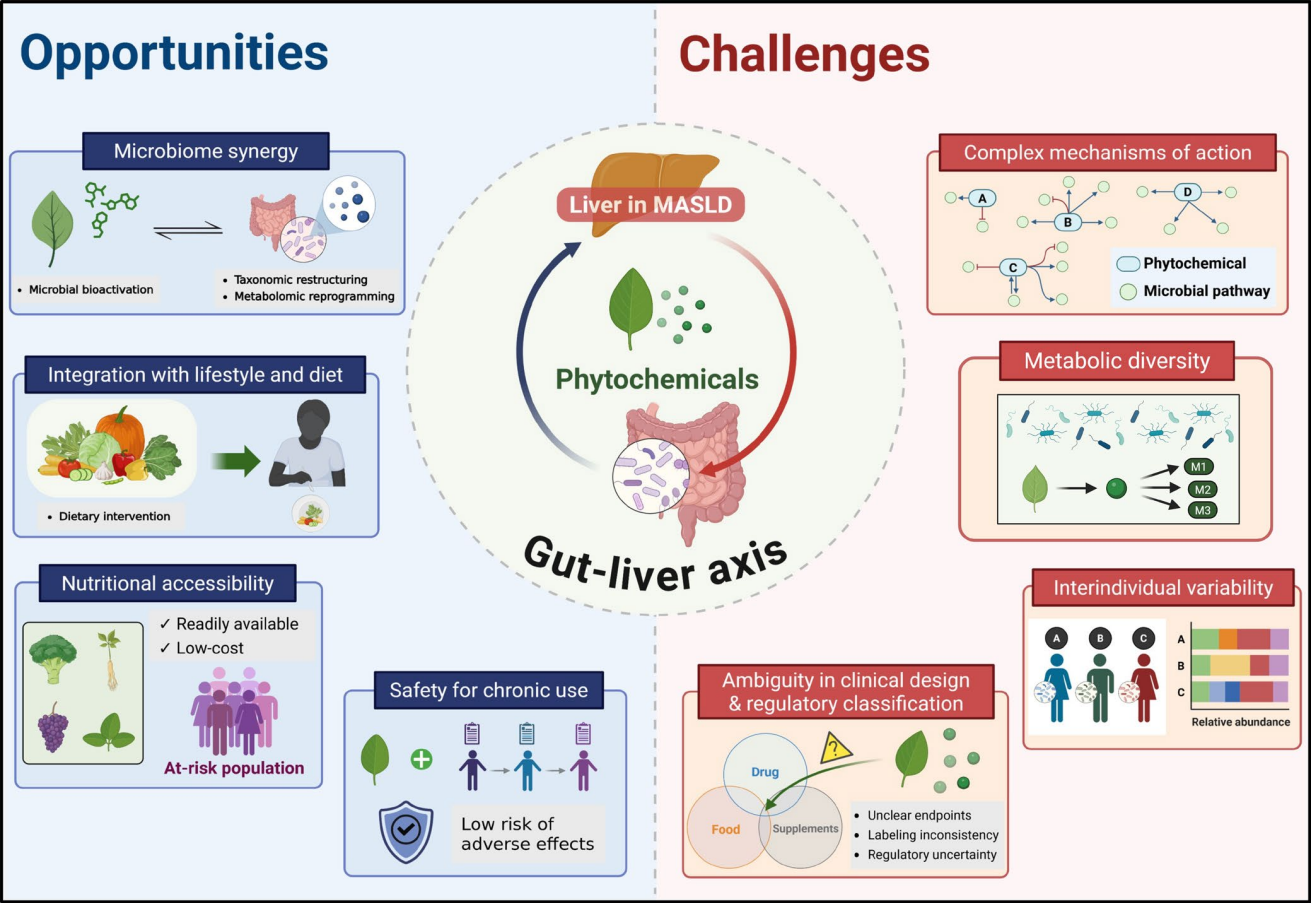
Opportunities and challenges in utilizing phytochemicals in MASLD therapy. Phytochemicals present unique therapeutic opportunities for MASLD by targeting the gut–liver axis through microbiota‐dependent bioactivation and modulation of the gut microbiome and its metabolites. Additional advantages include their compatibility with dietary and lifestyle interventions, broad nutritional accessibility, and suitability for long‐term use. Nonetheless, their clinical translation is challenged by complex and pleiotropic mechanisms, metabolic diversity, interindividual variability in microbiome responses, and regulatory uncertainty. Figure was created with BioRender.com.

Phytochemicals represent a promising therapeutic class uniquely suited to modulate the gut–liver axis in MASLD. Their appeal lies in several distinct pharmacological and translational advantages (Figure 3):

#### 4.1.1 Microbiome Synergy

Many phytochemicals depend on microbial transformation to become bioactive, establishing a relationship in which the gut microbiota serves as a metabolic activator. Furthermore, as a therapeutic target, unlike conventional drugs that typically act on a single pathway, phytochemicals engage multiple host–microbiome axes, enhancing microbial diversity, increasing SCFA production, modulating BA signaling via FXR/TGR5, and attenuating intestinal inflammation. This broad‐spectrum action is especially relevant for MASLD, a disease with complex and multifactorial pathology.

#### 4.1.2 Integration with Lifestyle and Diet

Phytochemicals naturally align with dietary patterns and behavioral strategies, offering a unique opportunity to embed microbiome‐targeted therapy into comprehensive lifestyle management.

#### 4.1.3 Nutritional Accessibility

Due to their origin in common foods and medicinal plants, phytochemicals are readily available and low‐cost, making them attractive options for early MASLD intervention or prevention in at‐risk populations.

#### 4.1.4 Safety for Chronic Use

Long‐term consumption of many phytochemicals has been validated by traditional use and clinical experience, making them suitable for sustained metabolic disease modulation with a low risk of adverse effects.

### 4.2 Remaining Barriers to Translating Phytochemicals into MASLD Therapy

Despite their therapeutic potential, several challenges continue to limit the clinical application of phytochemicals targeting the gut–liver axis in MASLD (Figure 3):

#### 4.2.1 Complex Mechanisms Complicate Quantification

Phytochemicals often act through multiple overlapping pathways, modulating microbial composition, BA metabolism, inflammatory cascades, and barrier integrity simultaneously. This pleiotropy makes it difficult to isolate specific effects or define precise pharmacodynamic markers for efficacy.

#### 4.2.2 Metabolic Diversity

Phytochemicals could be susceptible to extensive biotransformation by microbial enzymes (Zhang et al. 2026), which can alter their original structures and produce a wide array of metabolites with variable or unknown activity. These metabolic processes introduce interindividual variability, making reproducibility across individuals and the standardization of dosing major challenges.

#### 4.2.3 Interindividual Microbiome Variability

The efficacy of microbiome‐targeting phytochemicals depends heavily on a person's baseline gut microbial profile. Individuals with different microbial compositions may metabolize or respond to the same compound differently, leading to inconsistent outcomes in clinical studies unless patient stratification is incorporated.

#### 4.2.4 Ambiguity in Clinical Design and Regulatory Classification

Phytochemicals often straddle the boundaries between food, supplements, and pharmaceuticals. This creates uncertainty in trial endpoints, labeling standards, and approval processes, particularly when aiming to treat or prevent chronic diseases like MASLD. Without clear regulatory pathways, translational progress remains slow.

These barriers underscore the need for more precise, mechanism‐informed, and personalized approaches to unlock the full clinical value of phytochemicals in MASLD.

### 4.3 Future Research Directions

To overcome the key barriers limiting the clinical translation of phytochemicals in MASLD, future research must directly address the unique pharmacological and biological complexity of these compounds. First, the challenge of multi‐pathway actions and unclear dose–response relationships requires trials that integrate mechanistic biomarkers, such as changes in SCFA levels, BA profiles, or microbial taxa, with clinical endpoints. Mechanism‐linked study designs will make it possible to quantify effects across pathways rather than isolating a single target. Second, the metabolic complexity arising from both host and microbial metabolism necessitates advanced analytical approaches to trace the fate of phytochemicals in the host–microbiome system and identify functionally relevant compounds or metabolites. Third, to resolve interindividual variability, stratified trial designs based on baseline microbiome features, such as dominant taxa or functional gene profiles, should be employed to distinguish responders from non‐responders and guide personalized use. Lastly, clearer trial frameworks and regulatory definitions are needed to support phytochemical‐based interventions that straddle drug and nutritional domains. Collaborative efforts between clinical researchers, regulatory agencies, and industry can help develop standardized criteria for safety, efficacy, and labeling. By confronting these translational barriers directly, the field can advance toward precision‐guided, microbiome‐informed applications of phytochemicals in MASLD.

Seaweed provides a diverse range of phytochemicals whose relevance to MASLD remains insufficiently characterized, particularly in relation to possible interactions with gut microbiome mediated pathways. Although the available studies are limited, preclinical research shows that compounds derived from seaweed can improve hepatic and metabolic features associated with MASLD together with changes in gut microbial composition (Wang et al. 2021; Zhang et al. 2021). Clinical evidence, including research on dietary seaweed intake (Li et al. 2021) and clinical interventions using fucoxanthin (Bermont et al. 2025), a carotenoid present in brown seaweeds, has described improvements in hepatic or metabolic markers among individuals with MASLD, although these clinical investigations did not evaluate gut microbiome outcomes. Taken together, these findings indicate that seaweed‐based phytochemicals merit further investigation in MASLD, especially with regard to their potential interactions with the gut microbiome.

## 5 Conclusions

Phytochemicals offer a unique and promising avenue for MASLD therapy by targeting the gut–liver axis through natural, multitarget mechanisms. Their ability to modulate microbial composition, enhance beneficial metabolites, and attenuate inflammation aligns well with the multifactorial nature of MASLD. While current evidence from preclinical and clinical studies highlights their potential, key challenges, including mechanistic complexity, metabolic diversity, interindividual microbiome variability, and regulatory ambiguity, must be addressed. Future research that integrates mechanistic insights with clinical outcomes, applies multi‐omics profiling, and adopts stratified trial designs will be essential to fully realize the therapeutic value of phytochemicals. With the right framework, phytochemical‐based strategies may become viable, safe, and personalized interventions for the growing global burden of MASLD.

## Author Contributions

J.I.S. designed the review structure, conducted the literature search, selected key data, prepared and edited the manuscript, and created the table and figures. S.M.K. selected key data and created the table. H.H.Y. secured funding, designed the review structure, prepared and edited the manuscript, and provided supervision.

## Funding

This work was supported by National Research Foundation of Korea (RS‐2023‐00217123).

## Conflicts of Interest

The authors declare no conflicts of interest.

## Data Availability

The data that support the findings of this study are available on request from the corresponding author. The data are not publicly available due to privacy or ethical restrictions.
